# Axial electrokinetic trapping of anisotropic particles

**DOI:** 10.1038/s41598-019-39224-z

**Published:** 2019-02-26

**Authors:** Filip Strubbe, Bavo Robben, John Puthenparampil George, Íngrid Amer Cid, Filip Beunis, Kristiaan Neyts

**Affiliations:** Electronics and Information Systems Department and Center for Nano and Biophotonics, Ghent University, Technologiepark 126, 9052 Gent, Belgium

## Abstract

Anti-Brownian electrokinetic trapping is a method for trapping single particles in liquid based on particle position measurements and the application of feedback voltages. To achieve trapping in the axial direction, information on the axial particle position is required. However, existing strategies for determining the axial position that are based on measuring the size of the first diffraction ring, theory fitting, advanced optical setups or pre-determined axial image stacks are impractical for anisotropic particles. In this work, axial electrokinetic trapping of anisotropic particles is realized in devices with planar, transparent electrodes. The trapping algorithm uses Fourier-Bessel decomposition of standard microscopy images and is learning from the correlation between applied voltages and changes in the particle appearance. No previous knowledge on the particle appearance, theory fitting or advanced optical setup is required. The particle motion in the trap and the influence of screening of the electric field on this motion are analyzed. The axial trapping method opens new possibilities for measuring properties of anisotropic or isotropic particles and forces acting on such particles.

## Introduction

In recent years anti-Brownian electrokinetic trapping has become a valuable tool in the study of individual particles and inter-particle interactions in liquid^1–9^. Electrokinetic trapping essentially uses real-time electrical feedback based on particle position measurements to largely eliminate the particle’s Brownian motion. Particle trapping is used in the first place to study single particles for prolonged times while avoiding diffusion out of the measurement volume. Secondly, with trapping one can also avoid undesired interactions with interfaces during a measurement. And thirdly, trapping can be used to characterize the diffusion coefficient, *D*, and electrophoretic mobility, *μ*, of particles in real-time by analyzing the small deviations of the particle from the trap position^5^. In such a way, prolonged measurements of individual particles can reveal detailed information and dynamics that cannot be obtained by ensemble-averaged measurements. Since its first implementation, called the Anti-Brownian ELectrokinetic (ABEL) trap^1^, electrokinetic trapping has been applied to single fluorescent molecules^5,7^ and to fluorescent nanoparticles^6,8^. In most cases the electrokinetic trap is two-dimensional to trap a particle near a point in the plane of focus, while axial confinement is achieved mechanically by creating a thin channel. The particle position in the transverse plane is measured either using microscopy images or confocal laser scanning. But also full three-dimensional electrokinetic trapping has been realized in order to avoid particle interactions with the interfaces^6,8^. Axial trapping, perpendicular to the plane of focus, requires a much more challenging measurement of the axial position (*z*-position) of a particle.

Many strategies have been explored to determine the axial position of particles. For the case of standard microscopy images, one approach is to extract specific features from the images such as the distance from the particle center to the first bright diffraction ring and to create a calibration curve relating this distance to the axial position^6,10,11^. In astigmatic imaging, the axial position is inferred from the ellipticity of the particle image^8,12^. Another frequently encountered method is the use of a pre-determined look-up-table of images from which the axial position of a similar particle can be determined by interpolation^13^. In the case of holographic illumination, images of spherical particles and even of doublets and triplets can be fitted with Mie-scattering theory to extract the axial position at the cost of computational time^14–16^. Information on the axial position can also be obtained at the cost of a more complicated optical setup, for example with 3D orbital tracking of single nanoparticles^17^ or other methods^18–24^.

From the above it is clear that several interesting methods for axial particle tracking and axial trapping exist that are suitable for systems of well-defined particle shapes or sub-wavelength particles with well-defined optical images. However, in some situations involving polydisperse samples or anisotropic particles larger than the diffraction limit, it may not be possible to use a look-up-table, to fit the images with theory sufficiently fast to achieve real-time trapping or to use laser scanning schemes. For these cases the question arises if a more general algorithm can be developed to achieve electrokinetic trapping based on standard microscopy images.

In this work, axial electrokinetic trapping of anisotropic particles such as doublets and triplets as well as spherical particles in water is demonstrated without requiring look-up-tables, theory fitting or other particle-specific methods. The algorithm is based on standard microscopy images and can be applied to typical anisotropic particles that produce *z*-dependent images and that are electrically charged. Here, axial trapping is achieved by using devices consisting of two planar glass plates covered by homogeneous transparent electrodes to generate a one-dimensional electric field in the *z*-direction. The presented method opens new possibilities for trapping and characterizing particles in lab-on-a-chip devices with microscale electrode geometries, for measuring forces on anisotropic particles and for characterizing electrokinetics and Faradaic reactions at the water-electrode interface.

## Results

### Algorithm for axial trapping

To achieve robust axial electrokinetic trapping of anisotropic particles an algorithm is developed with two key features. Firstly, a series expansion of each particle image matrix *I* centered around the intensity centroid is calculated using a set of 2-dimensional polar Fourier-Bessel functions (see Methods section). The result of the image decomposition is a set of *N* Fourier-Bessel coefficients *B*_*n*,*m*_ that can be represented by a single point ***P*** in an *N*-dimensional space ${{\mathbb{R}}}^{N}$. This projection onto a space of reduced dimension has the advantage that electrical feedback can be based on distances between points rather than on two-dimensional images. Secondly, in each running experiment the trapping algorithm learns in real-time from the observed correlation between applied voltages and the resulting changes in the Fourier-Bessel coefficients. A pure axial displacement of a particle (along the *z*-axis) moves the point ***P***, representing the Fourier-Bessel coefficients, along a one-dimensional curve in ${{\mathbb{R}}}^{N}$. The distance measured along this curve is automatically a monotonic function of the *z*-position, and the application of a positive or a negative voltage will induce a drift motion shifting the point ***P*** in opposite directions along the curve. Such voltage-correlated displacements in ${{\mathbb{R}}}^{N}$ provide all the necessary information to achieve particle trapping near a certain point on the curve or, equivalently, at a certain *z*-position. This principle remains valid also for anisotropic particles. It is precisely for its ability to easily obtain a monotonous function of the *z*-position and its flexibility when applied to anisotropic particles, that such image decomposition is used here. The trapping algorithm is elaborated first for the case of spherical particles and then for the case of anisotropic particles.

Let us consider an isotropic, spherically symmetrical particle with electrophoretic mobility *μ*. Decomposition of particle images into *N* Fourier-Bessel coefficients *B*_*n*,*m*_ results, in the absence of measurement noise, in points ***P*** on a curve ***C***(*z*) in ${{\mathbb{R}}}^{N}$ with normalized tangent vector ***T***(*z*) = (∂***C***(*z*)/∂*z*)/|∂***C***(*z*)/∂*z*| depending on the axial particle position *z*. Since the particle images are expected to be symmetrical around the centroid, coefficients *B*_*n*,*m*_ with *n* ≠ 0 are zero and can be ignored in the analysis. Images are acquired at a frame rate $f=1/{\rm{\Delta }}t$ and points ***P***_*i*_ are acquired at times ${t}_{i}=(i-1){\rm{\Delta }}t$. The firstly acquired particle position ***P***_1_ at time *t* = 0 (at the beginning of the experiment) is chosen as the trapping destination: ***P***_*target*_ ≡ ***P***_1_. As soon as a displacement from this target position is measured (for *i* > 1), a voltage

1 $$\,{V}_{i}=-\,k({{\boldsymbol{P}}}_{i}-{{\boldsymbol{P}}}_{{\rm{target}}})\cdot {\widehat{{\boldsymbol{T}}}}_{i}$$

is applied, with *k* > 0 the feedback strength and ${\widehat{{\boldsymbol{T}}}}_{i}$ an estimator of the normalized tangent vector ***T***(*z*_*target*_) for *μ* > 0 or of −***T***(*z*_*target*_) for *μ* < 0. Note that the voltage with a given index is applied with a delay $\rho {\rm{\Delta }}t$ after the point with the same index has been measured. Initially the tangent vector at the trapping destination ***T***(*z*_*target*_) is unknown, so for *i* = 1 a first estimate ${\widehat{{\boldsymbol{T}}}}_{1}$ is chosen to be a random vector. The estimator ${\widehat{{\boldsymbol{T}}}}_{i}$ is improved every time step for *i* > 1 according to:

2 $${\widehat{{\boldsymbol{T}}}}_{i}=\frac{{\sum }_{j=\,{\rm{\max }}(i-W,2)\,}^{i}\,{\rm{sgn}}(\rho {V}_{j-2}+(1-\rho ){V}_{j-1})({{\boldsymbol{P}}}_{j}-{{\boldsymbol{P}}}_{j-1})}{|{\sum }_{j=\,{\rm{\max }}(i-W,2)\,}^{i}\,{\rm{sgn}}(\rho {V}_{j-2}+(1-\rho ){V}_{j-1})({{\boldsymbol{P}}}_{j}-{{\boldsymbol{P}}}_{j-1})|}$$

where the delay time in the electrical feedback is assumed to be smaller than Δ*t*, such that 0 ≤ *ρ* ≤ 1. A running average with a time window *W*Δ*t* is used by taking the summation from *j* = max(*i* − *W*, 2) to *j* = *i*. Equation (2) is based on the presumed correlation between the position differences ***P***_*j*_ − ***P***_*j*−1_ and the voltages *V*_*j*−1_ and *V*_*j*−2_ applied in the previous time steps. This correlation between voltages and displacements is especially clear when evaluating a pure drift motion for a particle with electrophoretic mobility *μ* exploring a curve ***C***(*z*) which is a line with $|{\rm{\Delta }}z|=\alpha |{\rm{\Delta }}{\boldsymbol{P}}|$:

3 $${{\boldsymbol{P}}}_{j}-{{\boldsymbol{P}}}_{j-1}=\frac{{z}_{j}-{z}_{j-1}}{\alpha }{\boldsymbol{T}}=\frac{\mu }{\alpha }{\int }_{{t}_{j-1}}^{{t}_{j}}E(t)dt\,{\boldsymbol{T}}=\frac{\mu {\rm{\Delta }}t}{\alpha d}[\rho {V}_{j-2}+(1-\rho ){V}_{j-1}]{\boldsymbol{T}}$$

while assuming a delay time $\rho {\rm{\Delta }}t$, a proportional field response according to *E* = *V*/*d* (where *V* is the applied voltage and *d* is the distance between the electrodes), Brownian displacements that are small compared to field-induced motion and small measurement errors on the points ***P***_*i*_. Under these assumptions sgn(*ρV*_*j*−2_ + (1 − *ρ*)*V*_*j*−1_)(***P***_*j*_ − ***P***_*j*−1_) is, for every value of *j*, a vector proportional to ***T*** if *μ* > 0 or to −***T*** if *μ* < 0. As a result, ${\widehat{{\boldsymbol{T}}}}_{i}$ as given in equation (2) will indeed be an estimator of sgn(*μ*)***T***(*z*_*target*_). Independent of the sign of the particle mobility (which is a priori unknown), the feedback voltage according to equation (1) will trap the particle near the target destination. In many practical situations where some of these assumptions are only partially fulfilled, equation (2) still provides a good estimator for ***T***. By considering a linear relation $|{\rm{\Delta }}z|=\alpha |{\rm{\Delta }}{\boldsymbol{P}}|$ in the trapping region, the trap strength can be characterized by the dimensionless parameter *K* = |*μk*Δ*t*/*αd*|, which represents the fraction of the displacement from the target destination that is counteracted by drift during each time step. Once the tangent vector is optimized, one may choose to fix this vector.

The described algorithm for axial electrokinetic trapping can also be applied to particles with anisotropy in shape (e.g. doublets, rod-like particles, …) or in composition (e.g. Janus particles). Again, it is assumed that the anisotropic particle has a net charge, leading to electrophoretic motion in the presence of a field. Note that for particles with polarizable surfaces such as metallic particles or Janus particles having a metallic hemisphere, besides the electrophoretic force, also additional translational or rotational forces related to induced-charge phenomena must be considered^25,26^. In this work the focus is on dielectric anisotropic particles for which forces related to induced charges are small compared to diffusion and electrophoresis, as is evidenced from the absence of particle alignment with the field or drift in the (*x*, *y*)-plane.

Let us first consider anisotropic particles that are axisymmetric with symmetry axis at an inclination angle *θ* ∈ [0, *π*] with respect to the *z*-axis, and at an azimuth angle *φ* ∈ [0, 2*π*] in the transverse plane. The center of mass can be chosen to mark the particle *z*-position. Similar as above, we choose to restrict the image decomposition to *N* Fourier-Bessel coefficients *B*_*n*,*m*_ with *n* = 0, such that rotation of the particle around the *z*-axis has no effect on the resulting Fourier-Bessel coefficients. Therefore, the Fourier-Bessel coefficients are independent of *φ* and only depend on the angle *θ* and the axial position *z*. In other words, axisymmetric particles are optically characterized by a two-dimensional surface ***S***(*z*, *θ*) in the space ${{\mathbb{R}}}^{N}$. Suppose that the trapping experiment is started by setting a reference position ***P***_*target*_ in the Fourier-Bessel coefficient space, corresponding to the axial position *z*_*target*_ and the orientation *θ*_*target*_. The vector $\widehat{{\boldsymbol{T}}}({z}_{target},{\theta }_{target})$ established by equation (2) will in good approximation be tangential to the curve ***C***(*z*, *θ* = *θ*_*target*_) at this reference point. Again, this tangent vector can be fixed after optimization. Considering equation (1) for the feedback voltage, the particle will then become trapped at the intersection of ***S***(*z*, *θ*) with the plane (with dimension *N*−1) through ***P***_*target*_ and orthogonal to $\widehat{{\boldsymbol{T}}}({z}_{target},{\theta }_{target})$. This means that the particle is free to rotate to a different inclination angle *θ*_*i*_, where the particle is trapped along a different curve ***C***(*z*, *θ* = *θ*_*i*_). And, since not all the points in this intersection necessarily correspond to the same axial position *z*_*target*_, the particle may move up and down slightly as it rotates around.

For anisotropic particles which are not axisymmetric, the Fourier-Bessel coefficients *B*_*n*,*m*_ with *n* = 0 now depend on the *z* position and two angles: the inclination angle *θ* of a chosen vector ***N*** that is fixed to the particle and the azimuthal angle *ξ* for rotation around ***N***. In general, the particle now explores a volume ***V***(*z*, *θ*, *ξ*) in ${{\mathbb{R}}}^{N}$. The same trapping algorithm as described above for axisymmetric particles can be used here for axial trapping. And again, during rotation the trapping algorithm may cause the particle to move up or down slightly while rotating, if the intersection of the plane through *P*_*target*_ perpendicular to ${\widehat{{\boldsymbol{T}}}}_{n}$ and the volume ***V***(*z*, *θ*, *ξ*) corresponds to different axial positions.

### Experimental results for isotropic particles

**Figure 1:**
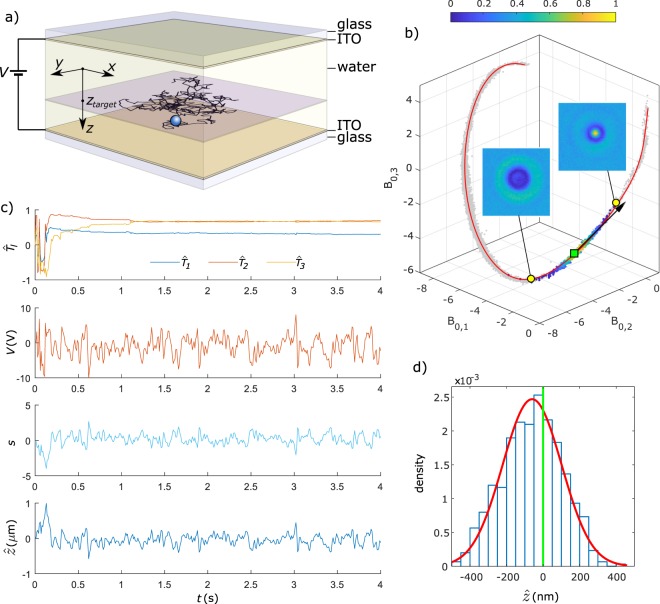
Principle of axial electrokinetic trapping demonstrated for a 1 μm spherical polystyrene particle in water. (**a**) Devices with transparent electrodes (ITO) on glass are used for axial trapping, with the microscope objective positioned below the bottom substrate. By applying feedback voltages *V* a single particle is trapped near the plane at *z* = *z*_*target*_ (Note that the *z*-axis is oriented downwards). (**b**) Fourier-Bessel decomposition of images of a free particle leads to coefficients (grey points) scattered along a curve $\hat{{\boldsymbol{C}}}(z)$ (red line). The inset shows images corresponding to two data points (yellow circles). When the trap is activated the density plot (colored data points) indicates that the particle stays close to the target destination (green square). (**c**) A detail of the time-dependency of the vector components ${\widehat{{\boldsymbol{T}}}}_{l}$ with *l* = 1, 2, 3, *V*, *s* and $\hat{z}$ is shown, where the trap is activated at *t* = 0. After about 1 s the tangent vector $\widehat{{\boldsymbol{T}}}$ is aligned with the curve ***C***(*z*) at *z* = *z*_*target*_ (black arrow in (b), the length of the vector is increased to 4 for clarity). (**d**) Histogram of $\hat{z}$ during 11.88 s of trapping (1188 data points) showing a standard deviation of 180 nm and an average position shifted −53 nm with respect to the target destination at $\hat{z}$ = 0.

The axial electrokinetic trapping of a 1 μm polystyrene bead in water is demonstrated in Fig. 1. Supplementary Movie 1 shows a similar particle axially trapped for 10 s. A device is used with planar, transparent electrodes (ITO) separated by a distance *d* = 77 μm (see Fig. 1a and Methods). This geometry allows to generate a homogeneous electrical field. By applying voltages according to equations (1) and (2) with ${\rm{\Delta }}t=0.01$ s and *ρ* = 1 a single particle is trapped near a plane at the axial position *z*_*target*_. This means that the particle remains free to move in the transverse plane parallel to the *x* and *y* axes. The microscopy stage is moved automatically in the (*x*, *y*)-plane to keep the particle within the field of view. The image decomposition is restricted to three Fourier-Bessel coefficients *B*_0,1_, *B*_0,2_ and *B*_0,3_. Figure 1b shows that without trapping (grey data points) the particle explores the curve ***C***(*z*) (red line). When the trap is activated at *t* = 0 s with *k* = 4 V, a density plot of the Fourier-Bessel coefficients (colored points) confirms that the particle stays close to the target destination (green square). The three components of the tangent vector estimator $\widehat{{\boldsymbol{T}}}$ are shown in Fig. 1c, illustrating that optimization is achieved after about 1 s. The average vector $\widehat{{\boldsymbol{T}}}$ after *t* = 1 s is plotted in Fig. 1b (black arrow) and is as expected approximately tangential to the curve. In all experiments a moving window with *W* = 100 is used to optimize $\widehat{{\boldsymbol{T}}}$. Also shown in Fig. 1c are the time dependencies of the feedback voltage *V*, the arc coordinate *s* of points projected on the curve ***C***(*z*) and the estimated axial position $\hat{z}$ (see Methods). The histogram of $\hat{z}$ in Fig. 1d reveals a distribution with $\langle \hat{z}\rangle =-\,53$ nm and ${\sigma }_{\hat{z}}=180$ nm. When assuming a linear response of the field to the applied voltage (*E* = *V*/*d*) an effective mobility value $\hat{\mu }$ is found by linear regression of:

4 $${\hat{z}}_{i+1}-{\hat{z}}_{i}=(1-\rho ){\rm{\Delta }}t\frac{{V}_{i}}{d}\mu +\rho {\rm{\Delta }}t\frac{{V}_{i-1}}{d}\mu +{\xi }_{i}$$

where the residuals *ξ*_*i*_ correspond to Brownian displacements. For the particle in Fig. 1, taking *ρ* = 1, the effective mobility is $\hat{\mu }$ = (−1.3 ± 0.2) × 10^−10^ m^2^V^−1^s^−1^. Note that this effective mobility can be much lower than the actual electrophoretic mobility due to screening of the electric field, as will be discussed further below. With this effective mobility a trap strength *K* = 0.32 is found using *α* = 210  nm obtained from the axial position calculation as explained in Supplementary Information. If the particle position is known from an independent measurement, the diffusion coefficient can be determined from the residuals *ξ*_*i*_ in equation (4). Here, the known diffusion coefficient is used to calibrate the position measurement (see Methods).

### Experimental results for anisotropic particles

**Figure 2:**
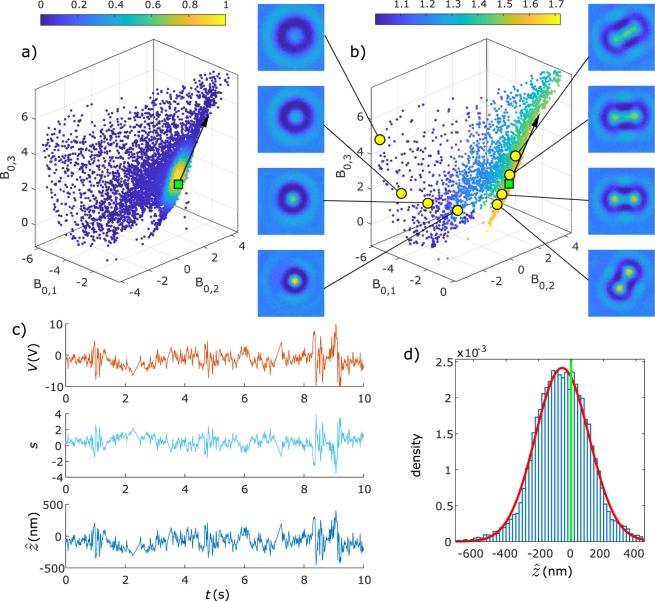
Axial electrokinetic trapping of a doublet of polystyrene particles trapped for 350 s seconds. (**a**) Density plot of 15494 Fourier-Bessel coefficients indicating trapping near the plane through the target destination (green square) and orthogonal to the tangent vector (black arrow, fixed after 2 s of optimization). (**b**) Plot with colors representing the ellipticity extracted from particle images. Eight particle images and their corresponding coefficients (yellow points) are shown. The images on the left and right correspond to *θ* = 0 and *θ* = *π*/2. respectively. (**c**) Detail of 10 s of trapping showing the time dependency of *V*, *s* and the axial position $\hat{z}$. (**d**) Histogram of $\hat{z}$ with standard deviation 165 nm and average position shifted −55 nm from the target position.

Figure 2 shows the trapping of a doublet formed by two contacting spherical polystyrene particles during 350 s using *k* = 3 V, again with ${\rm{\Delta }}t=0.01$ s and *ρ* = 1. Supplementary Movie 2 gives an example of a similar doublet axially trapped for 10 s. Since a doublet is an object with rotational symmetry around the axis connecting the two spheres, the coefficients *B*_0,1_, *B*_0,2_ and *B*_0,3_ depend only on the position *z* and the inclination angle *θ* of the axis of symmetry, forming a surface ***S***(*z*, *θ*). The density plot of the Fourier-Bessel coefficients in Fig. 2a confirms trapping near a plane orthogonal to $\widehat{{\boldsymbol{T}}}$ and through the reference position (green square). As the particle is freely rotating, a section of the surface ***S***(*z*, *θ*) is explored. The combination of the equal probability of all particle orientations, the fact that rotations around the z-axis do not affect the Fourier-Bessel coefficients here, and the way how the Fourier-Bessel coefficients depend on the inclination angle *θ*, results in a high density of coefficients near the particle orientation *θ* = *π*/2. Since most of the data is clustered near ***C***(*z*, *θ* = *π*/2), for most particle orientations a small displacement Δ*z* in the axial direction results in approximately the same displacement Δ*s* independent of the orientation *θ*. As a result, for most of the data points, $\hat{z}$ is still an acceptable estimator of the axial position (see Methods and Supplementary Information). To evaluate the particle orientation during trapping, the ellipticity of the particle images is plotted in Fig. 2b together with some characteristic images. On the left, four images are shown with ellipticity near 1 corresponding to *θ* = 0 (axis of symmetry parallel with *z*-axis) but at different axial positions. These particle images show a rotational symmetry and an increasing blurriness with decreasing *z*-position. On the right, four images with ellipticity near 1.7 are shown, corresponding to *θ* = *π*/2, again at different axial positions. Here, the connected spheres are nicely visible, again with increasing blurriness with decreasing *z*-position. The tangent vector $\widehat{{\boldsymbol{T}}}$ (black arrow) established after optimization is aligned well with data points corresponding to different *z*-positions but the same orientation *θ* ≅ *π*/2. Figure 2c shows a detail of the trapping data. The standard deviation of the particle position in Fig. 2d is ${\sigma }_{\hat{z}}$=165 nm and the offset is $\langle \hat{z}\rangle$ = −55 nm. The effective mobility obtained with equation (4) is $\hat{\mu }$ = (−4.77 ± 0.14) × 10^−11^ m^2^V^−1^s^−1^ and the corresponding trap strength is *K* = 0.11.

**Figure 3:**
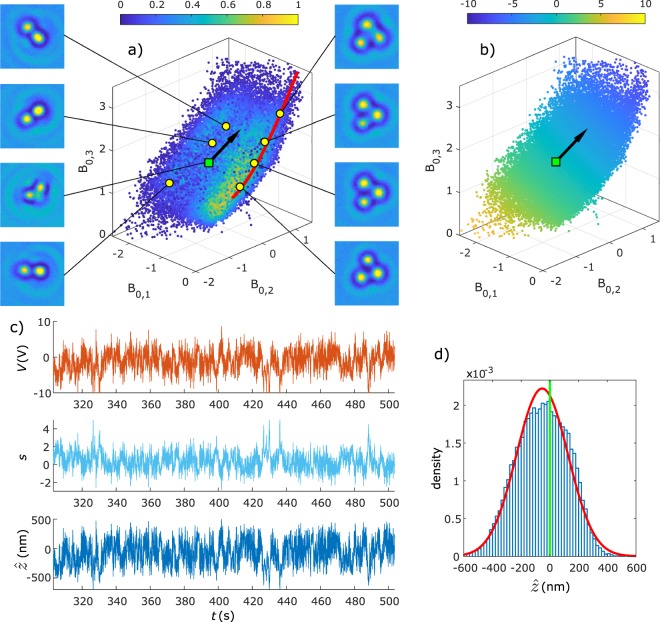
Axial electrokinetic trapping of a triplet of polystyrene particles trapped for about 760 s. (**a**) Density plot of Fourier-Bessel coefficients indicating trapping near the plane through the target destination (green square) and orthogonal to the tangent vector (black arrow, fixed after 2 s of optimization). Eight particle images and their corresponding coefficients (yellow/green points) are shown. (**b**) The same Fourier-Bessel coefficients with colors representing the feedback voltage applied after measuring each data point. (**c**) Detail of 200 s of trapping showing the time dependency of *V*, *s* and the axial position $\hat{z}$. (**d**) Histogram of $\hat{z}$ (34545 data points) with standard deviation 180 nm and average position shifted −53 nm from the target position.

Finally, a particle without axisymmetry is axially trapped for 760 s with *k* = 3 V, ${\rm{\Delta }}t=0.01$ s and *ρ* = 1, namely a triplet consisting of three polystyrene particles in a triangular configuration with ***N*** the unit vector normal to plane of the constituent particles (see Fig. 3). Supplementary Movie 3 gives an example of a similar triplet axially trapped for 10 s. Now the density plot of the Fourier-Bessel coefficients in Fig. 3a reveals a volume ***V***(*z*, *θ*, *ξ*), where *θ* is the inclination angle of ***N*** with respect to the *z*-axis, and *ξ* is the angle of rotation around the normal ***N***. Again, regions with a high density are visible, corresponding to particle orientations for which the Fourier-Bessel coefficients are relatively insensitive to deviations from that orientation. The red line corresponds to ***C***(*z*, *θ* = 0, *ξ*) for which *θ* = 0, the only inclination angle for which the Fourier-Bessel coefficients with *n* = 0 are independent of *ξ*. This red curve therefore indicates the general direction in which coefficients shift because of axial motion. The highest density of points is observed close to this curve. And as expected the vector $\widehat{{\boldsymbol{T}}}$ (black arrow) is approximately tangential to this curve. For all other inclination angles *θ* there is (in theory) a corresponding two-dimensional surface ***S***(*z*, *θ* ≠ 0, *ξ*), which is continuous for *ξ* ∈ [0, 2*π*] and which has a three-fold symmetry for rotation. Figure 3b shows the feedback voltage applied after measuring each data point. A detail of the trapping sequence is shown in Fig. 3c. The histogram of $\hat{z}$ in Fig. 3d deviates from a normal distribution, most likely due to dynamics of the field response. The data is used to calculate the effective mobility $\hat{\mu }$ = (−4.97 ± 0.13) × 10^−11^ m^2^V^−1^s^−1^, trap strength *K* = 0.08, standard deviation ${\sigma }_{\hat{z}}$ = 180 nm and offset $\langle \hat{z}\rangle$ = −53 nm.

### Analysis of field dynamics

In many implementations of electrokinetic trapping an electric field is generated by applying a voltage difference across reservoirs connected by a thin channel. The electric field in this thin channel is then sustained by a continuous electrical current and by Faradaic reactions at the electrode-liquid interface. Gas formation at the electrodes does not present a large problem since this occurs far from where the measurement is carried out in the thin channel. The electric field is then approximately equal to the applied voltage divided by the length of the microfluidic channel. In this work, planar, transparent electrodes are used to generate a one-dimensional electric field in the bulk of the liquid. Here, the electric field is not simply proportional to the applied voltage but is dynamic, depending on time and on the *z*-position. The underlying mechanism for these field dynamics is the accumulation of ions near the electrodes and Faradaic reactions occurring at the electrodes. In the presented experiments the particle motion is influenced by these field dynamics, but trapping is still possible. In other situations where the electric field is more strongly screened by accumulated ions within the feedback time, particle trapping can become difficult. Then, using a microfluidic channel to apply an axial field component, as used for example in Kayci *et al*.^6^, is a suitable alternative to achieve axial trapping.

**Figure 4:**
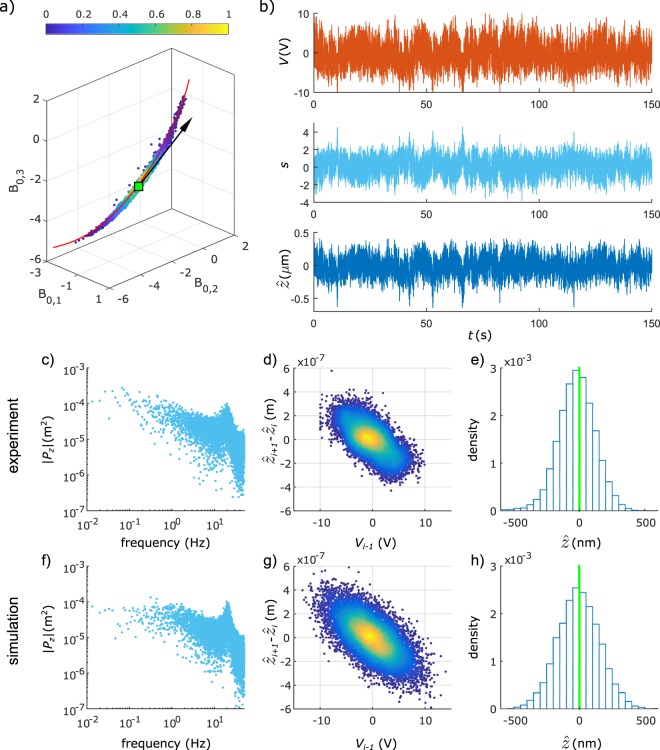
Detailed analysis of trapping data and field dynamics. A 1 μm spherical polystyrene particle is axially trapped during 151 s, resulting in 15082 data points. (**a**) Density plot of the Fourier-Bessel coefficients, with fitted curve $\hat{C}(z)$ (red line) and fixed tangent vector $\widehat{{\boldsymbol{T}}}$ after *t* = 2 s. (**b**) Complete trapping sequence showing *V*, *s* and $\hat{z}$ as a function of time. A detail of the first 4 s of trapping showing also the vector components ${\widehat{{\boldsymbol{T}}}}_{l}$ is shown in Supplementary Fig. S1. Experimental results and simulations are shown of the power spectrum of the particle *z*-position (**c**,**f**), the particle response to applied voltages (**d**,**g**) and the position histogram (**e**,**h**). The experimental distribution of $\hat{z}$ has a standard deviation 142 nm and an offset of −20 nm with respect to the target position.

Next, the effect of electric field dynamics on the particle motion are analyzed by using a large data set of a particle trapped for 150 s, also with ${\rm{\Delta }}t=0.01$ s and *ρ* = 1 (see Fig. 4). Figure 4a shows the density plot of the Fourier-Bessel coefficients during trapping, confirming stable trapping. Applying equation (4) to the trapping data in Fig. 4b results in an effective mobility $\hat{\mu }$ = (−2.01 ± 0.02) × 10^−10^ m^2^V^−1^s^−1^. The trap is relatively strong (*K* = 0.61, obtained with *k* = 3 V and *α* = 126 nm). Because of the feedback delay (*ρ* = 1) in combination with the large value of *K*, oscillations are visible in the plots of *V*, *s* and $\hat{z}$ as a function of time (see detail in Supplementary Fig. S1). To simplify the analysis, the vector $\widehat{{\boldsymbol{T}}}$ is fixed after optimization, at 2 s. In this experiment, when the running average of the voltage in a 1 s window exceeds 1 V, the feedback voltage is set to zero to avoid damage to the electrodes. As a result, narrow spikes are observed in the *z*-position when the particle drops more than expected. In Fig. 4c–e the experimental power spectrum of the particle position during trapping, the particle displacement *z*_*i* + 1_ − *z*_*i*_ versus applied voltage *V*_*i*−1_ and the position histogram are shown, which capture the essential dynamics of the trapped particle. To interpret these dynamics, simulations of a trapped particle are carried out with parameters similar as in the experiment of Fig. 4. The particle displacement in each iteration of the feedback loop due to electrophoresis, diffusion and gravity is simulated with the following equation:

5 $${z}_{i+1}-{z}_{i}={\int }_{{t}_{i}}^{{t}_{i+1}}\mu {E}_{bulk}(t)dt+{v}_{grav}{\rm{\Delta }}t+{\xi }_{i}$$

where *μ* is the particle mobility, *E*_*bulk*_(*t*) is the electric field experienced by the particle in the bulk, and where *ξ*_*i*_ are random Brownian displacements following a normal distribution with standard deviation $\sqrt{2D{\rm{\Delta }}t}$ with *D* = 4.29 × 10^−13^ m^2^s^−1^. The sedimentation velocity is ${v}_{grav}=\frac{4}{3}\pi {R}^{3}({\rho }_{particle}-{\rho }_{water})g/(6\pi \eta R)$, with particle radius *R* = 0.5 µm, viscosity *η* = 1.0 mPas, density difference *ρ*_*particle*_ − *ρ*_*water*_ = 50 × 10^3^ gm^−3^ and *g* = 9.81 ms^−2^. The feedback voltage *V*_*i*_ is updated using *k* = 3 V and *α* = 126 nm and assuming a linear trend $|{\rm{\Delta }}z|=\alpha |{\rm{\Delta }}{\boldsymbol{P}}|$:

6 $$\,{V}_{i}=-\,\frac{k}{\alpha }{z}_{i}$$

If it is assumed that there are no field dynamics such that the bulk field is *E*_*bulk*_(*t*) = *V*_*i*_/*d* for *t* between *t*_*i*_ and *t*_*i*+1_ and taking *μ* = −2.0 × 10^−10^ m^2^V^−1^s^−1^ equal to the experimental value of $\hat{\mu }$, the simulated power spectrum of the position is different than the experimental spectrum (see Supplementary Fig. S2). The shape of the simulated power spectrum below 10 Hz and the position and shape of the characteristic peak around 20 Hz are different. A much better agreement is obtained when including field dynamics according to an *RC*-network (see Supplementary Fig. S3). In this *RC*-network, *R*_*bulk*_ corresponds to the bulk resistance, *C*_*dl*_ corresponds to the double layer capacitance and *R*_*dl*_ determines the current leaking through the double layer due to Faradaic reactions at the electrode-liquid interface. The bulk field *E*_*bulk*_(*t*) = *V*_*bulk*_(*t*)/*d*_*bulk*_, with *d*_*bulk*_ ≅ *d*, now depends on the history of applied voltages. An analytical formula for the particle displacement and the voltage across the double layer in the case of such an *RC*-network is given in Supplementary Information. The parameters of the electric model are representative for applied voltages around 1 V, corresponding approximately to the average voltage amplitude in the trapping experiments. The parameters are *R*_*bulk*_ = 143 Ω, *C*_*dl*_ = 70 μF and *R*_*dl*_ = 286 Ω for an overlapping electrode area of 4 × 10^−4^ m^2^, corresponding to a characteristic screening time of 10 ms and a steady-state current which is 20% of the current expected in the absence of field screening. When choosing a mobility *μ* = −3.8 × 10^−10^ m^2^V^−1^s^−1^, the simulated trapping data results in an effective mobility $\hat{\mu }=-2.0\times {10}^{-10}$ m^2^V^−1^s^−1^ as is observed in the experiment. Figure 4f demonstrates that a satisfying agreement is obtained in the power spectrum, both in the position and shape of the peak at 20 Hz as in the frequency response below 10 Hz. The particle response to voltages in Fig. 4g and the position histogram in Fig. 4h correspond well to the experiment. It should be noted that this linear *RC*-network is a simplification of the actual physics which involve a voltage-dependent double layer capacitance and a voltage-dependent leakage current, as is observed in electrical current measurements (see Supplementary Information). By neglecting the voltage dependency and choosing fixed parameters the above analysis does not describe the particle motion in the trap accurately. Therefore, a more detailed analysis including the voltage-dependency of the field dynamics is required to extract the electrophoretic mobility of the trapped particle. Since such a detailed analysis is beyond the scope of this paper, we restrict to effective mobility values using equation (4). The bulk conductivity of the water in the trapping experiments (*σ* = 1.4 mSm^−1^) is much higher than for pure DI water (*σ* = 5.5 µSm^−1^), possibly due to ion contamination originating from the ITO electrodes. The lower electrophoretic mobility values compared to the average value of *μ* = −2 × 10^−8^ m^2^V^−1^s^−1^ obtained with a Zetasizer (see Methods) may be related to stronger retardation forces at these higher conductivities.

## Discussion

The experiments demonstrate that axial trapping of anisotropic particles using the presented algorithm can be achieved without requiring any look-up table, theory fitting or particle-specific methods for obtaining the *z*-position. Because of this flexibility, particles from polydisperse samples and a wide range of particles such as nanoparticles, blood cells and absorbing pigment particles can potentially be trapped without the need for individual calibration. Axial trapping is expected to work if the microscopy images are of sufficient quality, the electrical feedback is sufficiently fast, and screening of the electric field and DC forces are limited. For highly anisotropic particles such as Janus particles or rod-like particles with high aspect ratios problems can emerge if rotation alters the optical appearance such that (***P***_*i*_ − ***P***_*target*_) . $\widehat{{\boldsymbol{T}}}$ deviates too much from a proportionality with (*z* − *z*_*target*_). In such cases stable trapping may still be achievable by adjusting the algorithm, for example by implementing automatic adaptation of the target position to maximize image sharpness while the particle rotates. Trapping can also be achieved in non-uniform fields, if dielectrophoresis and other induced-charge effects are limited. Full three-dimensional trapping of particles can be achieved by using devices having also lateral electrode structures or orthogonal microfluidic channels similar as used by Kayci *et al*.^6^ or King *et al*.^8^ and by providing appropriate feedback in the (*x*, *y*)-plane. Devices with microscale electrode geometries as used in this work are useful for applications with low bulk conductivities, where a homogeneous field and low voltage differences are desired and limited sample volumes are available. However, at higher bulk conductivities screening of the electric field can become problematic for achieving trapping in such devices. Then, devices based on microfluidic channels can be used to avoid screening effects. In the presented experiments, only three Fourier-Bessel coefficients are selected, and the radius of the Fourier-Bessel functions and the feedback strength *k* are set manually. The algorithm can be further improved by including more coefficients or by automatically adjusting parameters. Offsets of the particle position with respect to the target position between −20 and −55 nm are observed. Even though small positive offsets can be expected due to the gravitational force (see Supplementary information), there can be larger offsets due to a combination of screening of the electric field and electrostatic or other forces acting on the particle in DC.

Particle trapping allows to carry out long experiments on a single particle in liquid. This can be an independent measurement as demonstrated by Kayci *et al*.^27^. Or, the trapping data itself can be collected and analyzed to measure particle properties with higher accuracy, to measure dynamics of particle properties, interactions with other particles or to measure forces acting on a particle. Analysis of particle properties relies on accurate knowledge of the particle position and the response of the electrical field to applied voltages. In this work the *z*-position is estimated by analyzing (pseudo-free) Brownian motion and the electrophoretic motion is analyzed using a simplifying *RC*-network, which limits the accuracy of the analysis. By using complementary methods to obtain the *z*-position and orientation (in real-time or in post-processing) and by using devices with a proportional field response, more accurate measurements can be carried out. For example, the tensors ***μ*** and ***D*** for anisotropic particles^16,28^ or femtonewton DC forces acting on trapped particles could be measured in such a way. In theory the accuracy by which a constant force can be measured is given by ${\sigma }_{F}=\sqrt{{q}_{eff}k{k}_{B}T/\alpha dN}$, where *q*_*eff*_ = 6*πηRμ*, *k*_*B*_*T* is the thermal energy and *N* is the number of sampled trapping positions. With parameters similar as in Fig. 4, using *N* = 15000 data points, the expected accuracy of a force measurement is 0.1 fN. Force sensing has also been demonstrated with optically trapped particles^29–32^. An advantage of electrokinetic trapping is that forces can also be measured on particles that cannot easily be optically trapped, such as high refractive index, highly scattering or absorbing particles. In the specific case of blood cells it is known that their weight and electrophoretic mobility are sensitive indicators for diseases^33–35^. Many efforts are being made to miniaturize devices for analysis of blood cells at the single cell level. Electrokinetic trapping could be an interesting way to measure the weight and electrophoretic mobility of blood cells at a reasonable throughput and sensitivity.

## Conclusion

Axial electrokinetic trapping of anisotropic particles has been demonstrated based on Fourier-Bessel image decomposition in combination with an algorithm that learns from the particle response to applied voltages. The benefit of the proposed algorithm is that it does not require prior knowledge about the particle appearance or absolute particle positions obtained from look-up tables or theory fitting. Axial trapping is demonstrated for singlet, doublet and triplets of polystyrene particles in water using devices with planar, transparent electrodes. In addition, the trapping data is analyzed to characterize the electrophoretic motion and field dynamics related to diffuse double layer charging and Faradaic reactions which affect the trap performance. The axial trapping algorithm can be used for the trapping and characterization of all kinds of anisotropic and isotropic particles in polydisperse samples and for the measurement of forces acting on them.

## Methods

### Sample preparation

Polystyrene particles of nominal diameter 1 μm are dispersed in water at low particle concentration such that only one particle is observed in the field of view during trapping. Doublets, triplets of polystyrene particles can be fabricated under controlled conditions^16,36^. Here, we used doublets and triplets that were spontaneously present in a dispersion of 1 μm polystyrene beads. Measurements on a more concentrated sample of polystyrene particles in DI water using a Malvern Zetasizer reveal a broad distribution of particle mobilities with average value −2 × 10^−8^ m^2^V^−1^s^−1^ and standard deviation 2 × 10^−8^ m^2^V^−1^s^−1^.

### Devices and microscopy

Devices are fabricated with two glass slides coated with transparent electrodes (ITO) separated by a distance *d* = 77 μm using UV curing glue with spacer beads (see Fig. 1a). Particles are imaged with a 100× oil immersion objective from the bottom side, where the ITO-coated glass is 170 μm thick. Standard Köhler illumination is incident from the top side where a ITO-coated glass is 1.1 mm thick. The Andor iXon EMCCD camera is triggered at 100 Hz. The focal plane is fixed at a position at least 10 μm away from the bottom and top interface. Particles are kept within the field of view by automatically adjusting the microscopy stage position in the transverse plane about once per second. Electrical feedback is given with NI USB-6259 BNC, with a delay of 10 ms after each acquired image, hence *ρ* = 1.

### Fourier-Bessel image decomposition

Image decomposition is carried out with a set of 2-dimensional polar Fourier-Bessel functions ${{\rm{\Psi }}}_{n,m}(r,\phi )=\frac{1}{\sqrt{2\pi {N}_{n}^{m}}}{J}_{m}({x}_{mn}r/a){e}^{im\phi }$ in the polar coordinates *r* and *φ*, using a radius *a* = 30 pixels (corresponding to 2.4 μm), as described in detail in Strubbe *et al*.^37^. These functions form an orthonormal set in the *r*-interval [0, *a*]. They are set equal to zero outside of the interval [0, *a*] and with zero-boundary condition at *r* = *a*. *J*_*m*_ is the *m*-th order Bessel function of the first kind with positive zeros *x*_*mn*_, and with ${N}_{n}^{m}={a}^{2}{J}_{m+1}^{2}({x}_{mn})/2$. Fourier-Bessel coefficients *B*_*n*,*m*_ correspond to the convolution of normalized particle images centered around the intensity centroid with the different Fourier-Bessel functions.

### Fitting 3D data

For spherical particles, an algorithm for fitting a B-Spline one-dimensional curve to 3-dimensional scattered data (red curves in Figs 1b and 4b) is developed based on Wang *et al*.^38^ and Pottmann *et al*.^39^. Notes from Yuri Pekelny and Gershon Elber are gratefully acknowledged. Data points, ***P***, having a set of Fourier-Bessel coefficients as components, are projected onto the fitted one-dimensional curve in 3D space using a minimal distance algorithm, resulting in the arc length *s* for each data point ***P***. For anisotropic particles, data points are projected onto the tangent vector $\widehat{{\boldsymbol{T}}}$ to obtain the value *s*.

### Axial position estimation

The particle axial position is estimated from statistical analysis of the particle position data and from a known value of *D*. In the case of isotropic particles, first the arc length *s* is calculated of the projection of Fourier-Bessel coefficients onto the fitted 3D curve ***C***(*z*). Since *s* is a monotonous function of *z*, the *z*-position is then estimated by using a calibration method based on the statistics of Brownian motion, as explained in Strubbe *et al*.^37^. In the case that only trapping data is available (no free Brownian motion), first pseudo-free particle displacements are calculated as explained by Cohen *et al*.^2^. In the case of anisotropic particles Fourier-Bessel coefficients are first projected onto the optimized and fixed tangent vector to obtain a measure for the axial position that is also denoted *s*, followed by the same procedure to estimate *z*. For singlets, doublets and triplets the theoretical values used for the rotational average of the diffusion coefficient are respectively *D* = 4.29 × 10^−13^ m^2^s^−1^, 3.07 × 10^−13^ m^2^s^−1^ and 2.78 × 10^−13^ m^2^s^−1^ ^16^. Details of the method, as well as an error analysis, are given in Supplementary Information and Supplementary Figs S4–8.

## Supplementary information

Supplementary Information

Movie 1

Movie 2

Movie 3

## Data Availability

The datasets generated and analyzed during the current study are available from the corresponding author on reasonable request.
