# Supplementary material for: Axial electrokinetic trapping of anisotropic particles
Source: Sci Rep. 2019 Feb 26;9:2806. doi: 10.1038/s41598-019-39224-z (PMC6391534; doi:10.1038/s41598-019-39224-z)
Supplement: Supplementary file 1 — Supplementary Information [file 41598_2019_39224_MOESM1_ESM.docx]

**Axial electrokinetic trapping of anisotropic particles**

**Filip Strubbe, Bavo Robben, John Puthenparampil George, Íngrid Amer Cid, Filip Beunis, Kristiaan Neyts**

**Supplementary information**

**Axial position estimation**For the particle in Fig.1, the mean-squared-displacements ${\Delta s}^{2}\left( s \right)$ corresponding to free Brownian motion in the axial direction are analyzed, where $s$ is the arc length of Fourier-Bessel coefficients projected onto the curve $\boldsymbol{C}(z)$. If only trapping data is available (for Figs. 2-4), the procedure is based on pseudo-free displacements, as is illustrated using **Supplementary Figures S4-S8** for the particle in Fig. 4. First the pseudo-free displacements (see **Supplementary Fig. S4**) are calculated by correcting the measured displacements $\Delta s$ (see **Supplementary Fig. S5**) for the electrokinetic feedback under the assumption of a linear field response ($E=V/d$) and using the effective mobility $\hat{\mu}$ [^1^]. Knowing that ${\Delta s}^{2}\left( s \right)$ (see **Supplementary Fig. S6**) should correspond to ${\Delta z}^{2}\left( s \right)=2D\Delta t$ a transformation formula $\hat{z}\left( s \right)=\pm\int\sqrt{2D\Delta t}/{\sqrt{\left\langle{\Delta s}^{2} \right\rangle(s)}}ds$ can be established through numerical integration (see **Supplementary Fig. S7**). The sign $(\pm$) can be inferred from inspection of microscopy images, and a constant can be added such that $\hat{z}(s=0)=0$ corresponds to the target destination. The value $\alpha$, appearing in the relation $\left| \Delta z \right|=$ $\alpha\left| \Delta\boldsymbol{P} \right|=\alpha\left| \Delta s \right|$ and in $K=\left| {\mu k\Delta t}/{\alpha d} \right|$, is calculated as $\alpha=\sqrt{2D\Delta t}/{\sqrt{\left\langle{\Delta s}^{2} \right\rangle(s_{target})}}$. The mobility calculated with equation (4) uses linear regression on the particle displacement versus the applied voltage (see **Supplementary Fig. S8**). For the case of anisotropic particles in Figs. 2 and 3, an estimation of $\hat{z}$ is calculated in a similar way, which is of sufficient accuracy to evaluate the trap. Here, $s$ and $\hat{z}$ have a different interpretation as for the case of an isotropic particle. For simplicity $s$ is chosen as the length of the vector $\boldsymbol{P}-\boldsymbol{P}_{target}$ projected onto the fixed vector $\hat{\boldsymbol{T}}$, which results only in an approximated estimator of $z$ near the target destination. The estimation of $\hat{z}$ is then carried out in the same way as before, namely by comparing the pseudo-free mean-square-displacements ${\Delta s}^{2}(s)$ with the expected mean-square-displacement ${\Delta z}^{2}=2D\Delta t$. If most of the trapping data is concentrated along a single curve with low curvature (as is the case in the experiments), then $\hat{z}$ will still be an acceptable estimator of the axial position.

**Electrical measurements**
Transient current measurements are carried out on the devices used for trapping. Different DC voltages are applied starting from the equilibrium situation at $V$=0 V. A bulk conductivity of about $1.3\times{10}^{-3}$ Sm^-1^ was obtained taking an approximated overlapping electrode area of $4\times{10}^{-4}$ m², corresponding to a bulk resistance $R_{bulk}=$143 $\Omega$. Below 1V, characteristic exponential screening is observed with a time constant of 1 ms. Around 1V this time constant increases to 10 ms. The steady state current after 1 s is low for values below 1 V but increases rapidly around 1 V to about 20% of the initial current. These persisting currents are ascribed to Faradaic reactions such as electrolysis with a typical threshold voltage around 1V. Above 3 V steady-state currents are practically at the same level as the initial current. These electrical measurements reveal, as expected, a voltage-dependency of the double layer capacitances and of Faradaic reactions at the electrodes.

***RC*-network**
In the simulations, an approximation is made by using a linear *RC*-network (see **Supplementary Fig. S3**), in which the double layers are represented by fixed capacitances and the Faradaic currents are proportional to the voltage across the double layers. The particle displacement due to drift, gravity and diffusion for the case of the RC-network is given by:

|  | $z_{i+1}-z_{i}=\frac{\mu V_{i-1}\rho\Delta t}{d_{bulk}}\left( 1-\frac{2}{\left( \frac{R_{bulk}}{R_{dl}}+2 \right)} \right)+\frac{2\mu\tau}{d_{bulk}}\left( V_{dl,i-1}-\frac{V_{i-1}}{\left( \frac{R_{bulk}}{R_{dl}}+2 \right)} \right)\left( e^{-{\Delta t}/\tau}-e^{-{\left( 1-\rho\right)\Delta t}/\tau} \right)+\frac{\mu V_{i}\left( 1-\rho\right)\Delta t}{d_{bulk}}\left( 1-\frac{2}{\left( \frac{R_{bulk}}{R_{dl}}+2 \right)} \right)+\frac{2\mu\tau}{d_{bulk}}\left( V_{dl,i}-\frac{V_{i}}{\left( \frac{R_{bulk}}{R_{dl}}+2 \right)} \right)\left( e^{-{\left( 1-\rho\right)\Delta t}/\tau}-1 \right)+v_{grav}\Delta t+\xi_{i}$ |  |
| --- | --- | --- |

where $\tau=R_{bulk}C_{dl}$. $V_{dl,i}$ is the potential difference across the capacitor $C_{dl}$, calculated according to:

|  | $V_{dl,i}=\left( V_{dl,i-1}-\frac{V_{i-1}}{\left( \frac{R_{bulk}}{R_{dl}}+2 \right)} \right)e^{-{\Delta t}/\tau}+\frac{V_{i-1}}{\left( \frac{R_{bulk}}{R_{dl}}+2 \right)}$. |  |
| --- | --- | --- |

**Position offset of a trapped particle due to gravity**Let us theoretically analyze the particle offset in the case of weak trapping such that $K\ll1$ and assuming a linear response of the electric field ($E=V/d$). Then, the motion of a trapped particle can be described in good approximation by a thermally excited overdamped oscillator in a harmonic potential with spring constant $k_{s}={q_{eff}k}/{\alpha d}$, where $q_{eff}$ is the effective particle charge defined by $\mu={q_{eff}}/{6\pi\eta R}$. The expected distribution of particle positions is then a normal distribution with variance $\sigma_{z}^{2}={k_{B}T}/{k_{s}}$ and mean $\left\langle z \right\rangle=0$. In the case that $K>0.1$ the experimental variance will be larger than this theoretical value, but for simplicity we continue in the above approximation. In the presence of an additional DC force $F$ in the *z*-direction, the average particle position will be shifted to $\left\langle z \right\rangle={F\alpha d}/{q_{eff}k}$. Now, let us consider the specific case of the gravitational force. For the particle in Fig. 4, taking $F=F_{g}=0.26$ fN for the gravitational force on a 1 μm polystyrene bead in water, $\alpha=126$ nm, $\mu=-2.0\times{10}^{-10}$ m²V^-1^s^-1^, $q_{eff}=12e$, $d=77$ μm, $k$=3 V, the expected offset by gravity is $\left\langle z \right\rangle=$0.4 nm. In the case that the DC field is screened the resulting offset can become larger.

**Error analysis**The error analysis can be split up into an error of the estimated axial position and an error of the estimated mobility with equation (4). The theoretical error on the axial position of a spherical particle obtained from the analysis of Brownian motion is analyzed in detail in Strubbe *et al.* [^2^]. In summary, the relative error on ${\Delta s}^{2}(s)$ scales with $(\sqrt{2/M})$ where $M$ is the number of measurements of ${\Delta s}^{2}$ in the interval around $s$. And, the error on the reconstructed $z$-position relative to a known reference position $z_{0}$ is $\sigma_{\Delta z} = \Delta z \sqrt{2/N_{1}}$, where $\Delta z = z - z_{0}$ and $N_{1}$ is the number of data points obtained in the interval between $z_{0}$ and $z$ assuming that they are evenly distributed over these intervals. For example, for the experiment of Fig. 4, within the range of -200 nm to +200 nm of the trapping destination (containing almost all the data) the chosen intervals of $\Delta s$=0.5 contain up to 3000 data points, corresponding to an accuracy of 3% on ${\Delta s}^{2}(s)$. The total number of data points in this range is about 10000. Therefore, theoretically the absolute error on the axial position across this range is in the order of $\sqrt{2/10000}\times$400 nm =6 nm (or about 1.5% error). In the case that a pseudo-free particle motion is extracted from trapping data, (which is the case for the particles in Figs. 2, 3 and 4), we have assumed an unscreened field response ($E=V/d$) in the reconstruction and an effective mobility $\hat{\mu}$. A simulation has been made to evaluate the error resulting from ignoring field dynamics for the case of the particle in Fig. 4. This simplification leads to an overestimation of Brownian motion by a factor 1.3, such that the estimation of the axial position should be corrected with a factor 1.3. For anisotropic particles additional errors arise from the projection of data onto a single line in Fourier-Bessel space. Since most of the data is clustered near a single curve in both cases (doublet and triplet) this effect is expected to be limited. For the doublet (Fig. 2), near the trapping destination about 1000 to 4000 data points are available in each interval of $\Delta s$ =0.2. The total number of data points is about 50000. If all data of Fourier-Bessel coefficients were scattered along a line, the absolute error over the range of 200 nm would be about 2 nm. But, since the data is in fact scattered along a surface the actual error will be more than 2 nm. A similar analysis can be made for the triplet (Fig. 3). However, absolute error margins cannot be given without independent knowledge on the actual particle position. And, in addition to these theoretical errors there are also errors due to image noise. For this reason, the obtained axial position $\hat{z}$ is an approximated estimator of the axial position. From the reconstructed particle position, linear regression with equation (4) is used to obtain an effective mobility value $\hat{\mu}$. The mobility values and their standard errors are as follows for the four measured particles: particle 1 (Fig.1): $\hat{\mu}=\left( -1.3\pm0.2 \right)\times{10}^{-10}$ m²V^-1^s^-1^, particle 2 (Fig.2): $\hat{\mu}=\left( -4.77\pm0.14 \right)\times{10}^{-11}$ m²V^-1^s^-1^, particle 3 (Fig.3): $\hat{\mu}=\left( -4.97\pm0.13 \right)\times{10}^{-11}$ m²V^-1^s^-1^, and particle 4 (Fig.4): $\hat{\mu}=\left( -2.01\pm0.02 \right)\times{10}^{-10}$m²V^-1^s^-1^. Note that the effective mobility can be different than the actual electrophoretic mobility if field dynamics are present, as is the case here.

**Supplementary Figures**


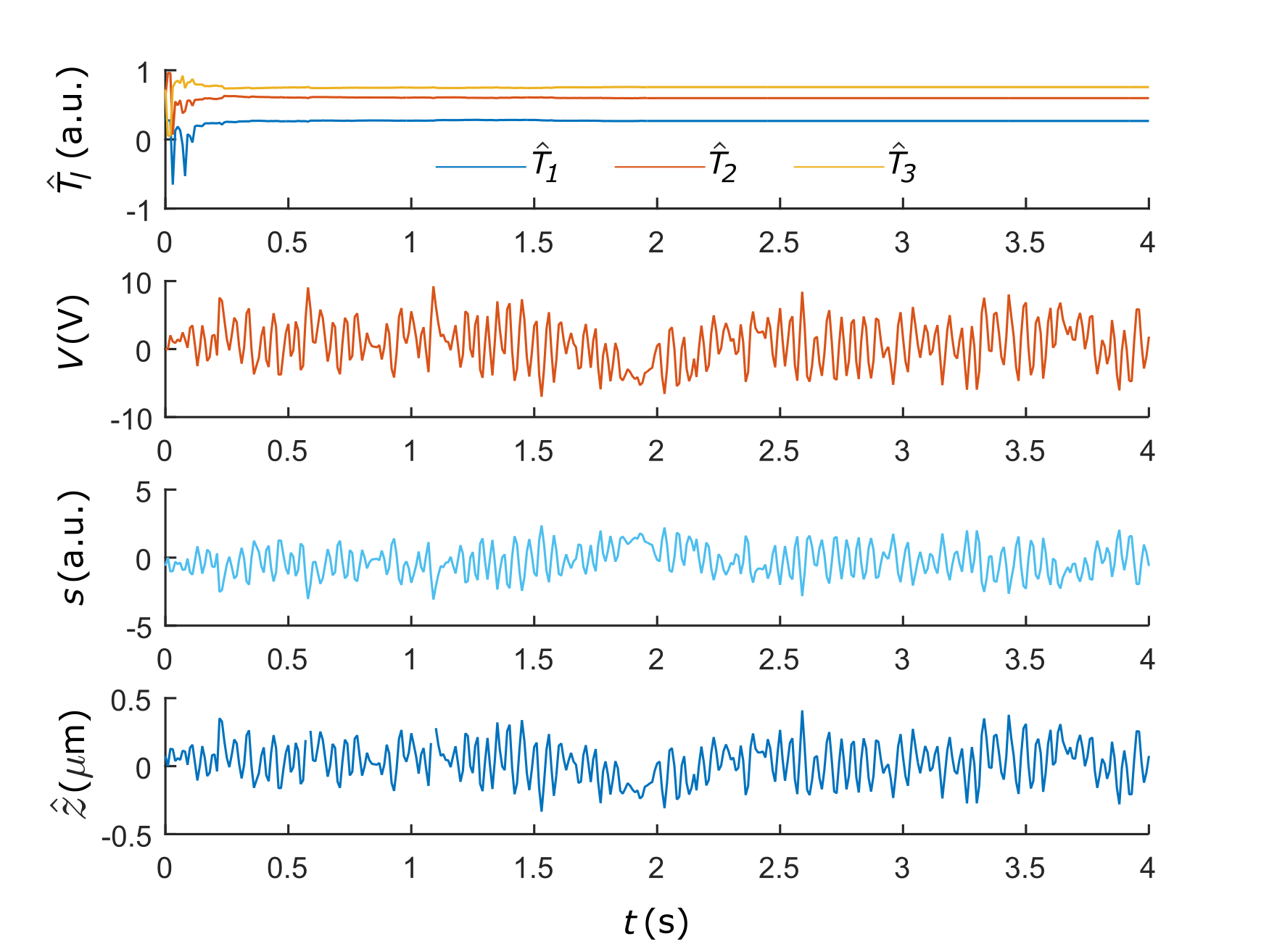


**Supplementary Figure S1. Detail of the first 4 s of axial trapping of the particle in Fig. 4.** The tangent vector components ${\hat{\boldsymbol{T}}}_{l}$ with $l=1,2,3$ show optimization in about 0.3 s. After 2 s the vector $\hat{\boldsymbol{T}}$ is fixed to simplify the analysis. $V$, $s$ and $\hat{z}$ as a function of time show oscillations because of the delayed feedback with $\rho=1$ and the strong feedback ($K=0.61$).


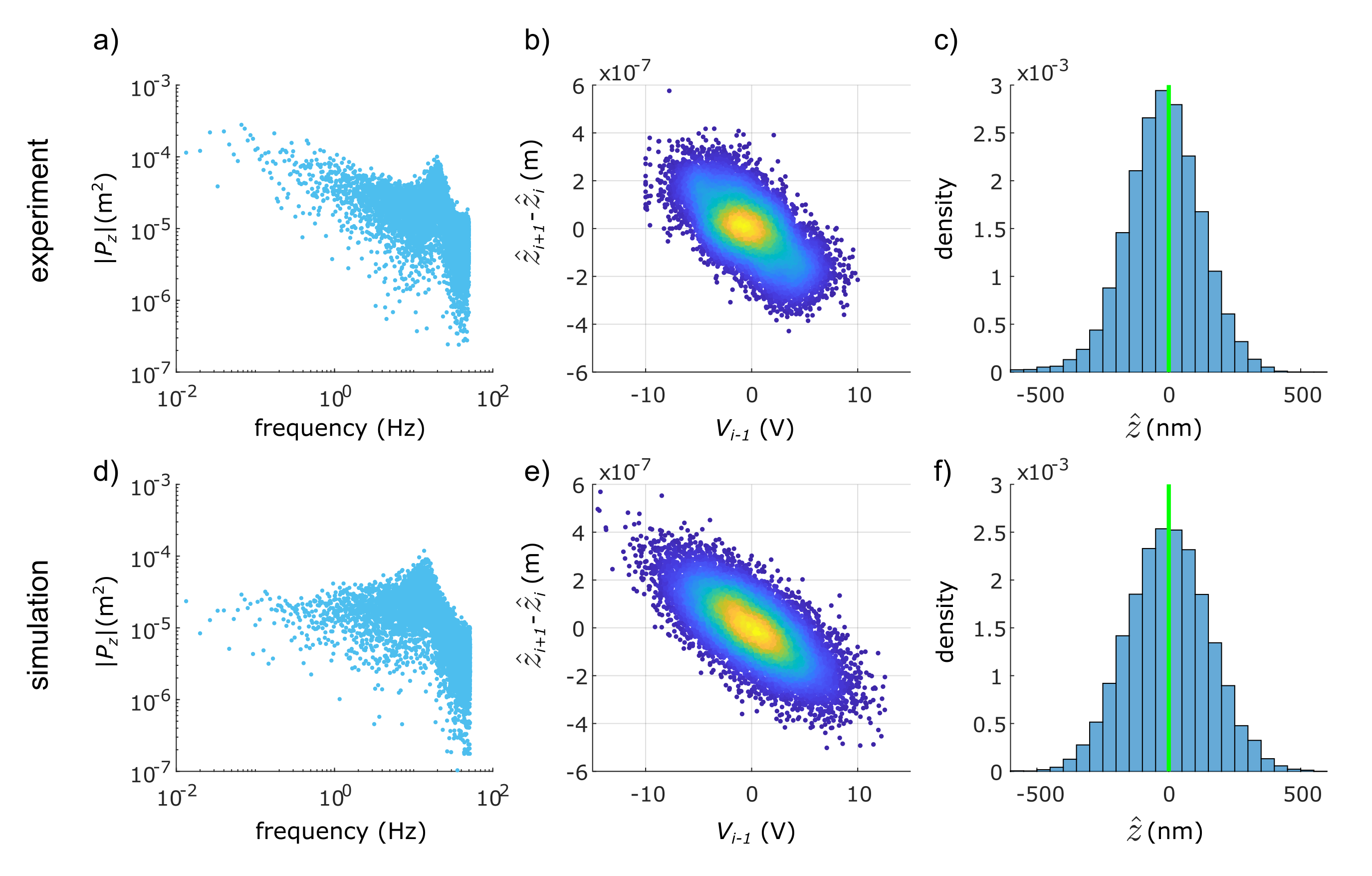


**Supplementary Figure S2. Comparison of the experiment of Fig 4, which is repeated here in (a-c), to simulations with a linear field response (d-f).** In the simulation, the mobility is $\mu=-2.0\times{10}^{-10}$m²V^-1^s^-1^, the same value as extracted from the experiment with equation (4). The other parameters are the same as used for Fig. 4, except that the field is simply determined by $E_{bulk}(t)=V_{i}/d$. The power spectrum of the particle position in (a) shows a characteristic decrease for frequencies below 10 Hz and a characteristic peak around 20 Hz. However, the simulated power spectrum of the particle position in (d) shows a much lower plateau below 10 Hz, and the position and shape of the peak are different compared to the experiment. The particle response to applied voltages (b, e) and the position histogram (c, f) are also shown. As expected from gravity, the simulated position histogram has an offset of about 0.4 nm, whereas the experimental offset is -20 nm.


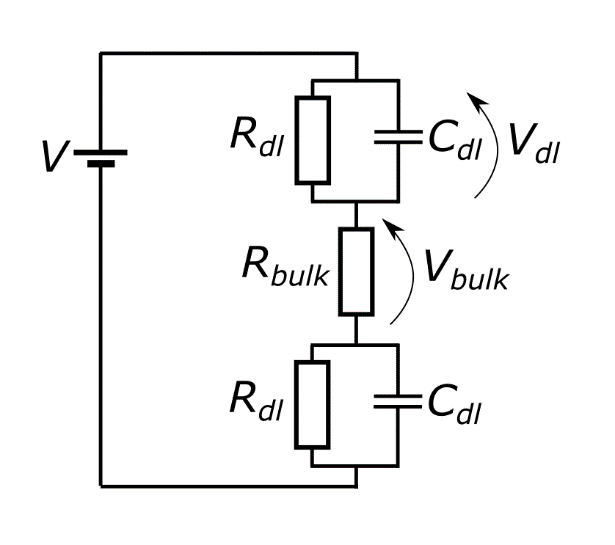


**Supplementary Figure S3. *RC*-network.** This electrical network provides an approximation of the internal electrodynamics in the water phase and Faradaic reactions at the electrode-liquid interface during trapping experiments. The particle is present in the bulk, where the electric field is homogeneous and given by $E_{bulk}=V_{bulk}/d_{bulk}$. Since the double-layers are much thinner than 1 $\mu$m, the bulk region can be approximated by the cell thickness: $d_{bulk}\cong d$. The two double-layers are represented by fixed capacitances $C_{dl}$. Current leakage through the double layers is modelled with resistances $R_{dl}$ in parallel to the double-layer capacitances.


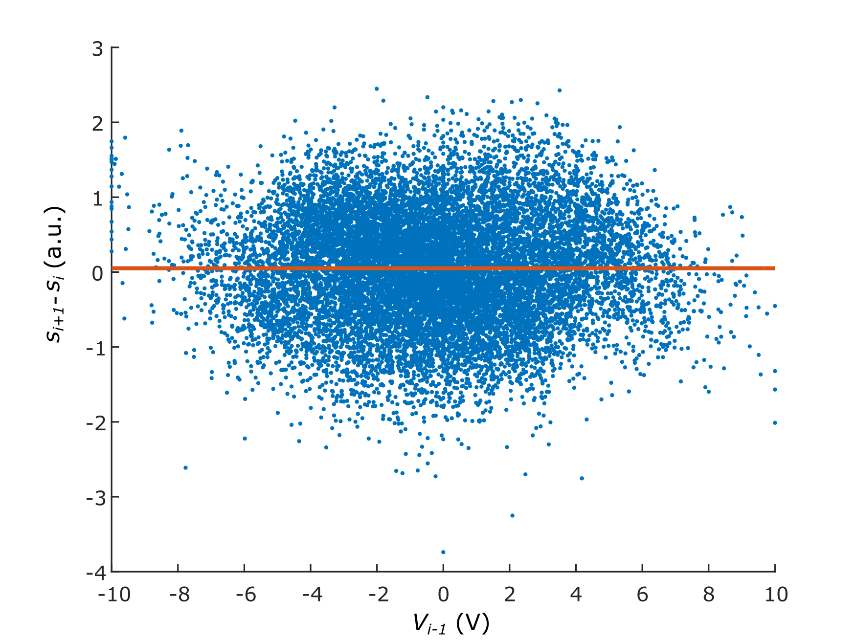


**Supplementary Figure S4. Voltage dependency of pseudo-free displacements of the particle of Fig. 4.** Pseudo-free displacements $s_{i+1}-s_{i}$ as a function of the applied voltage $V_{i-1}$ are obtained by subtracting the linear trend shown in Supplementary Fig. S5 (red line).


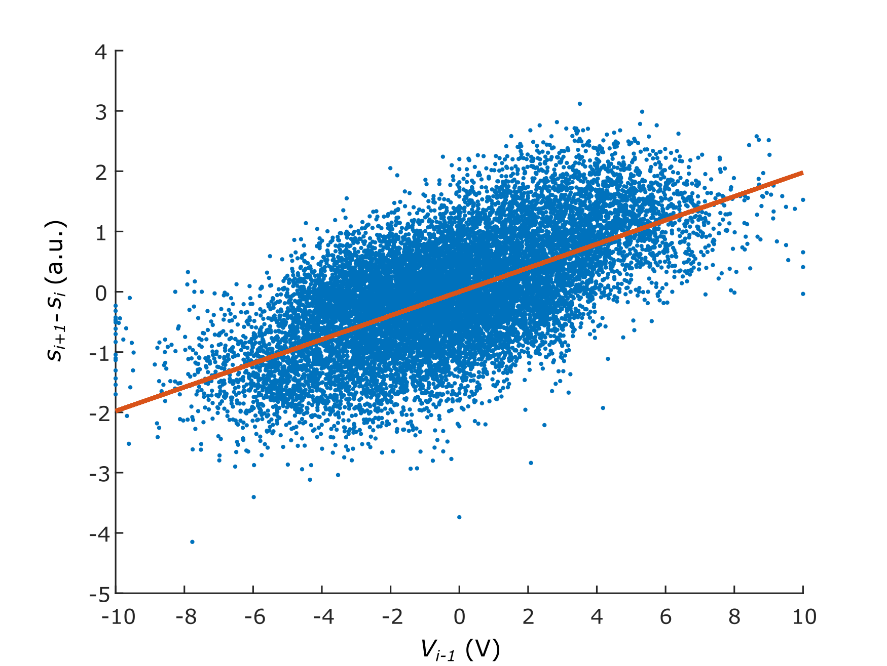


**Supplementary Figure S5. Voltage dependency of displacements of the particle of Fig. 4.** Displacements $s_{i+1}-s_{i}$ as a function of the applied voltage $V_{i-1}$, with linear regression (red line).


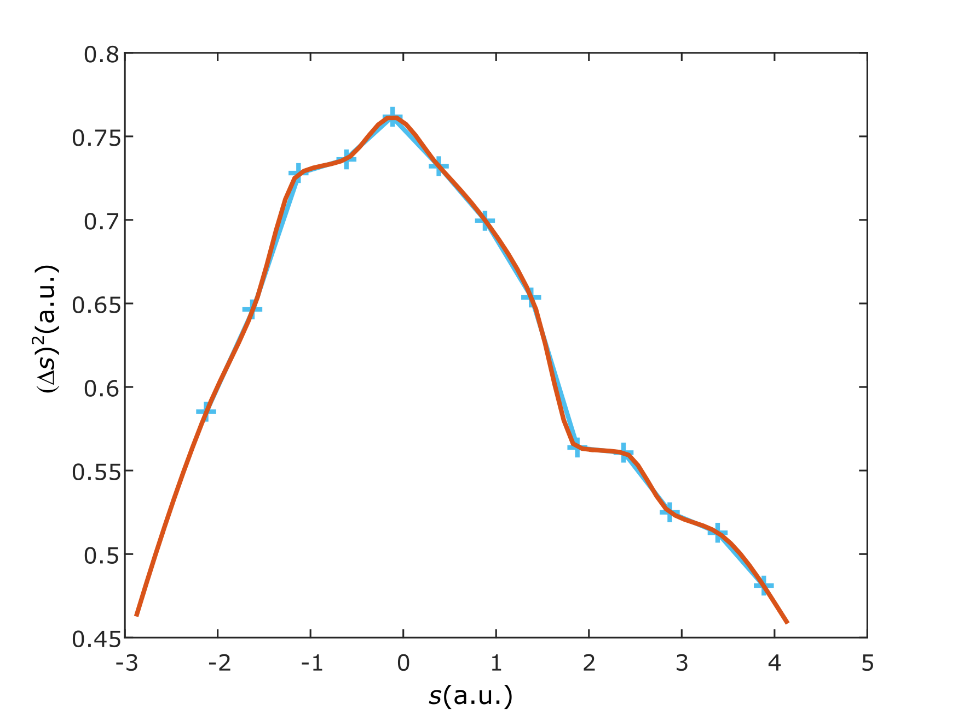


**Supplementary Figure S6. Mean-squared displacements for the particle of Fig. 4.** Mean-squared displacements $\left( \Delta s \right)^{2}$ are calculated in intervals along the $s$-axis (blue markers). A polynomial is fitted to the data (red curve).


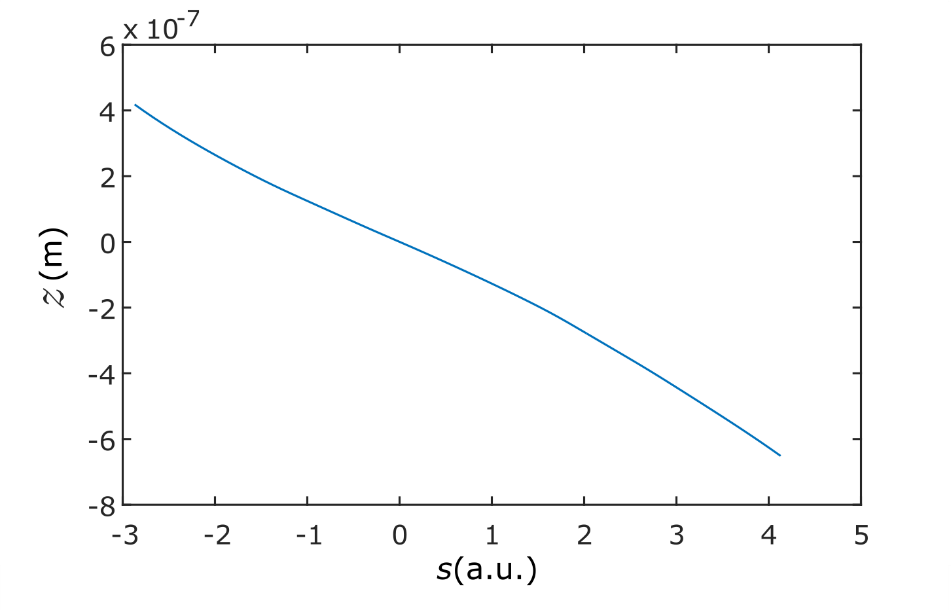


**Supplementary Figure S7. Position calibration of the particle of Fig. 4.** By integration of the square root of the polynomial in Supplementary Fig. S6 divided by $\sqrt{2D\Delta t}$, a conversion function is obtained between positions $s$ and the estimated axial position $\hat{z}$.


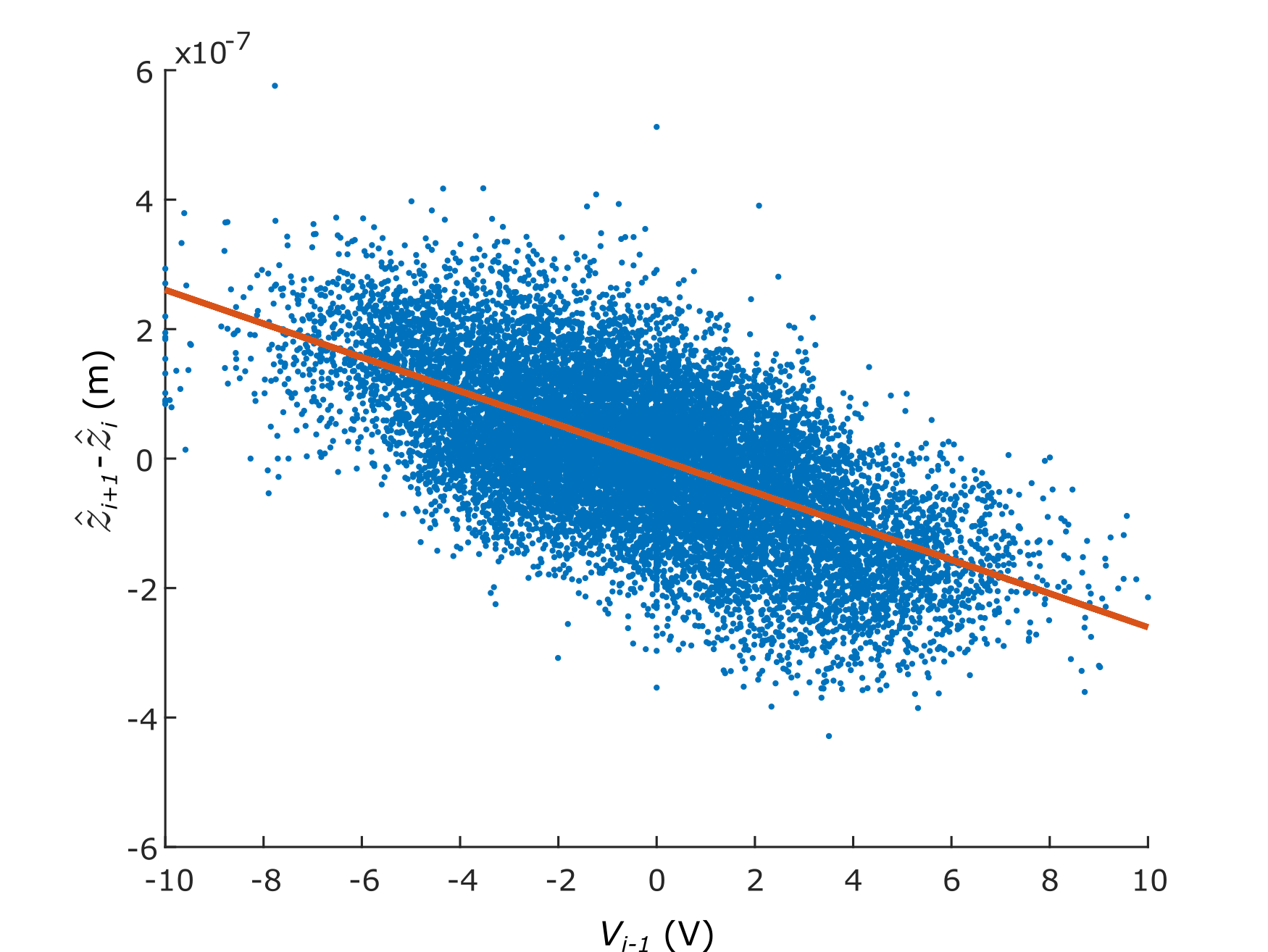


**Supplementary Figure S8. Mobility estimation assuming a linear field response.** Linear regression is applied to the particle displacements $\hat{z}_{i+1}-\hat{z}_{i}$ versus the applied voltage $V_{i-1}$ according to equation (4), to obtain an estimation for the electrophoretic mobility.

**Supplemetary Movies**

Movies 1, 2, 3 show the axial electrokinetic trapping respectively of a singlet, doublet and triplet of polystyrene beads in water. The actual duration is 10 s (1000 frames recorded at 100 Hz), but the frame rate of the movies is reduced for clarity. The particles are trapped near a plane parallel to the plane of focus, but are free to move around in this plane, and are free to rotate. To keep the particles within the field of view, the microscopy stage is adjusted automatically about every second, resulting visibly in occasional jumps of the particle (*x, y*)-position.

**References**

1 Cohen, A. E. & Moerner, W. E. Suppressing Brownian motion of individual biomolecules in solution. *P Natl Acad Sci USA* **103**, 4362-4365, doi:10.1073/pnas.0509976103 (2006).

2 Strubbe, F. *et al.* Characterizing and tracking individual colloidal particles using Fourier-Bessel image decomposition. *Opt Express* **22**, 24635-24645, doi:10.1364/Oe.22.024635 (2014).
